# Identification of an Immunodominant B-Cell Epitope in African Swine Fever Virus p30 Protein and Evidence of p30 Antibody-Mediated Antibody Dependent Cellular Cytotoxicity

**DOI:** 10.3390/v16050758

**Published:** 2024-05-10

**Authors:** Jessica C. G. Noll, Ruchi Rani, Salman L. Butt, Maureen Hoch Vieira Fernandes, Gabriela Mansano do Nascimento, Mathias Martins, Leonardo C. Caserta, Lina Covaleda, Diego G. Diel

**Affiliations:** Department of Population Medicine and Diagnostic Sciences, College of Veterinary Medicine, Cornell University, Ithaca, NY 14853, USAslb358@cornell.edu (S.L.B.); mhvf@stanford.edu (M.H.V.F.); mathias.martins@tvmdl.tamu.edu (M.M.); lcc88@cornell.edu (L.C.C.); lmc342@cornell.edu (L.C.)

**Keywords:** ASF, ASFV, African Swine Fever Virus, *CP204L*, p30

## Abstract

African Swine Fever Virus (ASFV) is a large dsDNA virus that encodes at least 150 proteins. The complexity of ASFV and lack of knowledge of effector immune functions and protective antigens have hindered the development of safe and effective ASF vaccines. In this study, we constructed four Orf virus recombinant vectors expressing individual ASFV genes *B602L*, -*CP204L*, *E184L*, and -*I73R* (ORFV^Δ121^-ASFV-*B602L*, -*CP204L*, -*E184L*, and -*I73R*). All recombinant viruses expressed the heterologous ASFV proteins in vitro. We then evaluated the immunogenicity of the recombinants by immunizing four-week-old piglets. In two independent animal studies, we observed high antibody titers against ASFV p30, encoded by *CP204L* gene. Using Pepscan ELISA, we identified a linear B-cell epitope of 12 amino acids in length (Peptide 15) located in an exposed loop region of p30 as an immunodominant ASFV epitope. Additionally, antibodies elicited against ASFV p30 presented antibody-dependent cellular cytotoxicity (ADCC) activity. These results underscore the role of p30 on antibody responses elicited against ASFV and highlight an important functional epitope that contributes to p30-specific antibody responses.

## 1. Introduction

African Swine Fever Virus (ASFV) is the sole member of the *Asfarviridae* family, in the genus *Asfivirus* and the only known DNA arbovirus.ASFV contains a large double-stranded DNA genome with approximately 170–190k base pairs. The icosahedral ASFV virion is highly complex, comprising six structural domains: the outer envelope; outer capsid; inner membrane; inner capsid; core shell; and the nucleoid, which contains the large DNA genome [1,2]. The virus is the causative agent of African Swine Fever (ASF), a transboundary hemorrhagic disease that poses a significant threat to the swine industry, as it causes up to 100% morbidity and mortality in domestic pigs (*Sus scrofa domesticus*) and wild boars (*Sus scrofa ferus*). The virus can also infect warthogs (*Phacochoerus africanus*) and bush pigs (*Phacochoerus porcus*); however, infection in these species is subclinical [3,4].

African Swine Fever was first reported over 100 years ago in Kenya [5]. Until the early 2000s, outbreaks of the disease were sporadic and mostly confined to the African continent. However, in 2007, ASF was reported in the Republic of Georgia, and from there, it spread throughout neighboring countries in Eastern Europe [6]. The virus was introduced in China in 2018, causing an unprecedented outbreak resulting in estimated losses of 43.46 million pigs (approximately 6.3% of the total number of pigs slaughtered in 2018) and causing major economic losses worldwide [7]. Following its introduction in China, the virus spread to several other countries in Asia, including Vietnam, Mongolia, Cambodia, the Democratic People’s Republic of Korea, the Lao People’s Democratic Republic, Myanmar, the Philippines, the Republic of Korea, Timor-Leste, Indonesia, Papua New Guinea, India, Malaysia, Bhutan, Thailand, Nepal, and Singapore, devastating the swine industry in the region [8]. Recently, the disease was reported in the Americas, on the Caribbean islands of Haiti and the Dominican Republic [6], and for the first time in Sweden, demonstrating that the virus continues to spread into new territories [9,10,11].

Currently, there are no efficacious treatments for ASFV, and available vaccines present safety concerns. Live attenuated vaccines (LAVs), derived from naturally attenuated strains or engineered via the deletion of genes associated with virulence, generally offer some level of protection against homologous challenge, conferring reduced clinical signs and viremia [12]. However, safety has been an issue with several LAVs, as vaccinated animals may become chronically infected with the vaccine virus, developing clinical signs such as fever, swollen joints, and hypergammaglobulinemia several months post-vaccination [12,13,14,15,16,17,18]. Subunit vaccines offer a safer approach to ASF vaccine development. However, the high complexity of the virus and the lack of well-characterized protective antigens are major hurdles that hinder the development of effective subunit vaccines [19]. 

This study aimed to assess the immunogenicity of four ASFV proteins: pB602L, p30, pE184L, and I73R. The pB602L, encoded by *B602L* gene, is a late non-structural protein of ASFV that functions as a chaperone for the folding of the major ASFV capsid protein p72, thus playing a regulatory role in the formation of the icosahedral viral capsid [20,21]. Low levels of antibodies against pB602L have been detected during ASFV infection around two weeks after the infection [22]. Protein E184L (pE184L), encoded by the *E184L* gene, is a virulence determinant of ASFV with approximately 22 kDa [23,24]. This protein was shown to be immunogenic [25] and capable of eliciting antibody and IFN-*γ* responses in pigs immunized with the pool of ASFV antigens, using vaccinia virus vectors [26]. 

The protein I73R (pI73R) is a structural protein of ASFV [23] that is expressed early in the viral replication cycle [27,28]. The *I73R* gene is nonessential for virus replication in vitro, but it contributes to ASFV virulence in vivo. This protein binds to cellular mRNAs and prevents their translation, therefore suppressing host innate immune responses and promoting viral replication. Animals inoculated with a *I73R* deleted mutant virus showed no clinical manifestations and cleared the infection after 28 days [29].

The phosphoprotein p30 encoded by *CP204L* (also known as p32) is a structural virion component of ASFV [23] with 30 to 32 kDa that is detected as early as 2 h post-infection (p.i.) in the membrane of cells infected with ASFV [30,31]. Although p30 is one of the most immunogenic proteins of ASFV capable of eliciting high levels of antibodies upon infection and/or immunization [30,31,32,33], the function of p30-specific antibodies and their role in ASFV immunity remain poorly understood. 

The results of an immunization study in pigs here revealed the immunodominance of p30 in comparison to the other three target proteins, B602L, E184L, and I73R. We then focused on characterizing the responses against ASFV p30. Using Pepscan ELISAs, we identified a twelve amino acid immunodominant B-cell epitope within one of the exposed loops of ASFV p30. The functional characterization of p30-directed antibodies demonstrated that they induce antibody-dependent cellular cytotoxicity (ADCC) and, thus, may contribute to ASFV immunity by mediating clearance of ASFV-infected cells. 

## 2. Materials and Methods

### 2.1. Cells and Viruses

Primary ovine fetal turbinate (OFTu) cells generated in house, porcine kidney (PK15), human embryonic kidney (HEK 293T), and baby hamster kidney cells (BHK21) (ATCC, Manassas, VA, USA) were cultured in minimum essential media (MEM) (Corning Inc., Corning, NY, USA) supplemented with 10% fetal bovine serum (FBS) (Omega Scientific, Tarzana, CA, USA), L-glutamine (2 mM) (Thermo Fisher Scientific, Waltham, MA, USA), penicillin (100 U/mL) (Thermo Fisher Scientific, Waltham, MA, USA), streptomycin (100 µg/mL) (Thermo Fisher Scientific, Waltham, MA, USA), amphotericin B (0.25 µg/mL) (Thermo Fisher Scientific, Waltham, MA, USA), and gentamicin (0.05 mg/mL) Corning Inc., Corning, NY, USA) and kept at 37 °C with 5% CO_2_. ORFV virus strain IA-82 [33], kindly provided by Dr. Daniel Rock from the University of Illinois at Urbana Champaign, was cultured in OFTu cells and utilized as the wild-type strain for the generation of recombinant virus vectoring ASFV genes. The 6-, 12-, 24-, and 96-well plates utilized in the experiments described below are from Greiner Bio-One (Monroe, NC, USA), and 100 mm cell culture dishes are from Corning Inc., (Corning, NY, USA).

### 2.2. Construction of Recombination and Expression Plasmids Encoding ASFV Genes

The ASFV genes *B602L*, *CP204L*, *E184L*, and *I73R* from genotype II strain Armenia/07 were chemically synthesized and cloned into a pUC57 backbone plasmid under the control of the vaccinia virus promoter I1L [34] with the addition of a flag tag (DYKDDDK) coding sequence at the 5′-end of each gene, and the restriction enzyme sites for PacI/SbfI and NotI/AscI at the 5′- and 3′-end of the gene sequences, respectively (Bio Basic, Markhamm, ON, Canada). The DNA fragments containing the I1L promoter and encoding the ASFV-flag tagged proteins were excised from the pUC57 backbone plasmid via restriction digestion and subcloned into a poxvirus recombination/transfer plasmid containing the left and right flanks of ORFV open reading frame 121 (*ORFV121*) [35] and the green fluorescent protein (GFP) reporter gene under control of vaccinia virus vv7.5 promoter, flanked by two LoxP sequences (locus of X(cross)-over in P1) (pUC57-ORFVΔ121-loxP-eGFP). Cloning of the ASFV genes into the pUC57-ORFVΔ121-loxP-eGFP recombination plasmid was confirmed by restriction digestion and gel electrophoresis analysis in 1% agarose gels.

Mammalian expression plasmids based on the pCAGGS plasmid were generated for ASFV *B602L*, *CP204L*, *E184L*, and *I73R* genes. For this, the ASFV genes were PCR amplified from the pUC57 plasmids using primers containing an HA epitope coding sequence, which was designed to swap the flag tag fusion epitope by the HA tag (YPYDVPDYA). Purified PCR amplicons were digested by their respective restriction enzymes and cloned into the pCAGGS plasmid. Cloning was confirmed via a restriction digestion analysis in 1% agarose gels.

### 2.3. Generation of Recombinant Viruses

The ASFV *B602L*, *CP204L*, *E184L*, and *I73R* genes were inserted into the *ORFV121* locus of the ORFV vector via homologous recombination. For this, OFTu cells were cultured in 6-well plates (5 × 10^5^ cells per well) for 24 h, infected with ORFV-IA82 at a multiplicity of infection (MOI) of 1, and incubated at 37 °C for 1 h. The inoculum was replaced with complete growth media, and cells were incubated for 2 h at 37 °C. The cells were then transfected with 2.5 µg/well of each recombination plasmid (pUC57-ORFVΔ121-ASFV-loxP-eGFP), using Lipofectamine 3000 reagent (Thermo Fisher Scientific, Waltham, MA, USA), following the manufacturer’s instructions. At 72 h post-infection/transfection, the cells and supernatant were harvested and subjected to three freeze and thaw cycles, and cell lysates were then used for recombinant virus selection via plaque assay.

Briefly, OFTu cells were cultured in 6-well plates and infected with 10-fold serial dilution of the infection/transfection cell lysate (10^−1^–10^−3^). After 1 h of incubation at 37 °C, the inoculum was removed, and the cells were overlayed with a 1:1 mixture of 2× complete growth media and 1% agarose (SeaKem GTC agarose, Lonza, Alpharetta, GA, USA). At 72 h post-infection, the plates were screened for GFP-positive plaques. Plaques were marked, picked, placed in sterile microcentrifuge tubes containing 250 µL of MEM, and stored at −80 °C. The following plaque assays were performed by infecting OFTu cells with 10-fold dilutions (10^−1^–10^−3^) of the selected plaques/clones, until wild-type virus plaques (GFP-negative) were no longer observed. The presence of ASFV genes and the absence of *ORFV121* were confirmed by PCR.

Once the recombinant ORFV-ASFV viruses were purified, the GFP reporter was removed from the virus genome using the CRE-LoxP system [36]. For this, OFTu cells were cultured in 6-well plates and transfected with 2.5 µg/well pBS-MCK-CRE plasmid (a gift from Ronald Kahn (Addgene plasmid # 12529)), utilizing Lipofectamine 3000 reagent (Thermo Fisher Scientific, Wiltham, MA, USA), following the manufacturer’s instructions. At 24 h post-transfection, cells were infected with ORFV^∆121^ASFV-GFP recombinants and incubated at 37 °C for 72 h. Cells and supernatant were collected and subjected to three cycles of freeze and thaw. The cell lysate was then used to infect new OFTu cells transiently expressing the Cre recombinase as described above. After three passages in Cre recombinase-expressing cells, the ORFV^∆121^ASFV-GFP recombinants were subjected to a plaque assay as described above. GPF-negative plaques were picked and subjected to additional rounds of plaque assays until GFP-positive plaques were no longer observed. The presence of ASFV genes and the absence of GFP were confirmed by PCR. Stocks of the different ORFV^∆121^ASFV recombinants were amplified, titrated in OFTu cells, and stored at −80 °C until further use. The integrity of ORFV sequences and correct insertion of ASFV gene sequences in the recombinant ORFV^∆121^ASFV viruses was confirmed by whole-genome sequencing, using a combination of Ligation Sequencing Kit and PCR Barcoding Expansion (Oxford Nanopore Technologies, ONT). Libraries were loaded onto a FLO-MIN106 R9.4 flow cell and sequenced using the Oxford Nanopore MK1C sequencing platform. 

### 2.4. Immunofluorescence

The expression of ASFV B602L, p30, E184L, and I73R proteins by the recombinant ORFV^∆121^ASFV viruses, as well as by the pCAGGS mammalian expression vectors, was assessed by immunofluorescence (IFA). OFTu cells were cultured in 24-well plates (2.5 × 10^5^ cells per well) for 24 h and infected with ORFV^∆121^ASFV-B602L, -CP204L, -E184L, and -I73R recombinant viruses (multiplicity of infection [MOI] = 1). HEK 293T cells were cultured in 24-well plates (2.5 × 10^5^ cells per well) for 24 h and transfected with pCAGGS-ASFV-B602L, pCAGGS-ASFV-CP204L, pCAGGS-ASFV-E184L, or pCAGGS-ASFV-I73R (2.5 µg of DNA), using Lipofectamine 3000 (Thermo Fisher Scientific, Waltham, MA, USA), following the manufacturer’s instructions. At 24 h p.i. or post-transfection, cells were fixed with 3.7% formaldehyde for 30 min at room temperature (RT) and rinsed with 1X phosphate buffered saline (PBS) (Corning Inc., Corning, NY, USA) three times. To determine intracellular or cell surface expression of ASFV proteins, cells were either permeabilized with 0.2% Triton X-100 (MP Biomedicals, Santa Ana, CA, USA) in PBS for 10 min at RT or left non-permeabilized and rinsed with 1X PBS three times. To detect the expression of the ASFV proteins, infected OFTu cells were incubated with Flag-tag monoclonal antibody (Sigma Aldrich, St. Louis, MO, USA), while transfected HEK 293T cells were incubated with HA-tag monoclonal antibody (GenScript, Piscataway, NJ, USA) diluted 1:250 in PBS 1% bovine serum albumin (BSA) (Fisher Scientific, Hampton, NH, USA) for 30 min, followed by three washes with PBS. Next, cells were incubated with goat anti-mouse IgG (H+L), Affinity Pure, and DyeLight^®^594 Conjugate antibody (ImmunoReagents Inc., Raleigh, NC, USA) diluted 1:250 in PBS 1% BSA for 30 min at RT. After three washes with 1X PBS, the cells were examined under a fluorescence microscope (Olympus CKX53, 20× magnification or Zeiss 710 Confocal, 40× magnification). 

### 2.5. Western Blot

OFTu cells were cultured in 6-well plates (5 × 10^5^ cells per well) for 24 h and infected with ORFV^∆121^ASFV-*B602L*, -*CP204L*, -*E184L*, and -*I73R* recombinant viruses (MOI = 5). At 48 h p.i., cells and supernatant were harvested and centrifuged at 6000× *g* for 5 min. The cell pellet was resuspended in 200 µL Mammalian Protein Extraction Reagent (M-PER, Thermo Fisher Scientific, Waltham, MA, USA) containing 1× Protease/Phosphatase Inhibitor cocktail (Cell Signaling Technology, Danvers, MA, USA). The cell lysate was sonicated three times for 8 s at 10% amplitude (Branson 450 Digital Sonifier), and protein concentration was measured using the Qubit Fluorometer (Thermo Fischer Scientific, Waltham, MA, USA). Approximately 100 µg of total protein was loaded into a 10% TGX polyacrylamide gel (Bio-Rad, Hercules, CA, USA) and subjected to electrophoresis at 80 V for 110 min. The resolved proteins were then transferred to nitrocellulose membranes using the Trans-Blot Turbo Transfer System and Trans-Blot Turbo RTA Midi 0.2 μm Nitrocellulose Transfer Kit (Bio-Rad, Hercules, CA, USA) according to the manufacturer’s instructions.

The membranes were blocked in Tris-buffered saline (TBS) (Research Products International, Mt. Prospect, IL, USA) containing 5% non-fat dry milk (Research Products International, Mt. Prospect, IL, USA) overnight at 4 °C and subsequently washed three times in TBS–0.2% Tween 20 (10% Tween 20 non-ionic detergent, Bio-Rad, Hercules, CA, USA) (TBST) for 5 min each time. The Flag epitope-specific monoclonal antibody (Sigma Aldrich, St. Louis, MO, USA) diluted 1:1000 in TBS 5% BSA was added to the membranes and incubated at RT for 1h, followed by three washing steps, as described above. Next, the membranes were incubated with the IRDye 800CW Goat Anti-Mouse IgG Secondary Antibody (LI-COR Biosciences, Lincoln, NE, USA), diluted 1:10,000 in TBS 1% BSA, for 1h at RT. After three washing steps, the membranes were imaged using the ChemiDoc MP Imaging System (BioRad, Hercules, CA, USA).

### 2.6. Replication Kinetics

OFTu and PK15 cells were infected with ORFV^∆121^ASFV-*B602L*, -*CP204L*, -*E184L*, and -*I73R* recombinant viruses (MOI = 0.1 and 10). The cells and supernatants were collected at 0, 6, 12-, 24-, 48-, and 72-hours p.i. and stored at −80 °C. Viral titers were determined at each time point by Spearman and Karber’s method [37], using limiting dilutions, and expressed as tissue culture infectious dose 50 (TCID_50_) per milliliter. 

### 2.7. Flow Cytometry

OFTu cells were seeded in 6-well plates (5 × 10^5^ cells per well) and infected with ORFV^∆121^ASFV-*B602L*, -*CP204L*, -*E184L*, and -*I73R* recombinant viruses (MOI = 5) (two wells per virus). At 48 h p.i., cells and supernatant were harvested and centrifuged at 500× *g* for 5 min at RT (Centrifuge 5810 R 15-amp version, Eppendorf, Framingham, MA, USA). The cell supernatant was discarded, and the pellets were resuspended in 300 µL of 3.7% formaldehyde (VWR, Radnor, PA, USA). After 30 min of incubation at RT, the cell suspension was transferred to three wells of a 96-well U-bottom plate, and the plate was centrifuged at 500× *g* for 5 min at RT. One well for each virus was permeabilized with 0.2% Triton-X in PBS for 10 min. Next, the plate was centrifuged (500× *g* for 5 min), and the supernatant was discarded. The Flag-tag antibody (Sigma Aldrich, St. Louis, MO, USA) diluted 1:250 in 1% FBS-PBS was added to the permeabilized and non-permeabilized cells (50 µL/well), while the third well was kept as unstained control. After 30 min of incubation, the plate was centrifuged at 500× *g* for 5 min at RT, and the supernatant was discarded. Goat anti-mouse IgG (H+L) and DyeLight^®^488 Conjugate antibody (ImmunoReagents Inc., Raleigh, NC, USA) diluted 1:250 in 1% FBS-PBS was added to permeabilized and non-permeabilized cells (50 µL/well). After 30 min of incubation, the plate was centrifuged at 500× *g* for 5 min at RT, and the supernatant was discarded. The cell pellet was resuspended in 200 µL of PBS, the cells were analyzed with an Attune NxT flow cytometer (Thermo Fisher Scientific, Waltham, MA, USA), and the data were analyzed using the FlowJo software v10.10 (BD Biosciences, Franklin Lakes, NJ, USA).

### 2.8. Animal Immunization Studies

The immunogenicity of the ORFV^∆121^ASFV-*B602L*, -*CP204L*, -*E184L*, and -*I73R* recombinant viruses was evaluated in pigs. Initially, eight four-week-old piglets were immunized with a pool of ORFV^∆121^ASFV-*B602L*, -*CP204L*, -*E184L*, and -*I73R* viruses containing 10^7.13^ TCID_50_ of each virus in 5 mL. The animals were immunized on day 0 and boosted on day 21 (Study 1). Serum samples were collected on days 0, 7, 14, 21, 28, and 35 post-immunization (p.im.), and the experiment was terminated on day 35 p.im. A second immunization study was conducted with the ORFV^∆121^ASFV-CP204L recombinant virus (Study 2). For this, six four-week-old piglets were immunized with 10^7.13^ TCID_50_ of ORFV^∆121^ASFV-CP204L on day 0 and boosted on days 14 and 28. Serum samples were collected on days 0, 14, 21, 28, 35, and 42 p.im., and the experiment was terminated on day 42 p.im. All animal studies were reviewed and approved by the Institutional Animal Care and Use Committee (IACUC) from Cornell University (Approval No. 2020-0093). 

### 2.9. Immunoprecipitation

HEK 293T cells were cultured in 100 cm cell-culture dishes (2 × 10^6^ cells per dish) for 24 h and transfected with 10 µg of plasmids pCAGGS-ASFV-*B602L*, -*CP204L*, -*E184L*, and -*I73R*, using Lipofectamine 3000 (Thermo Fisher Scientific, Waltham, WA, USA) according to the manufacturer’s instructions. At 48 h after transfection, cells were collected and centrifuged at 3000× *g* for 10 min. The cell pellet was resuspended in 200 µL of IP Lysis Buffer (Thermo Fisher Scientific, Waltham, WA, USA) containing 1X Protease/Phosphatase Inhibitor cocktail (Cell Signaling Technology, Danvers, MA, USA). After three cycles of freeze and thaw at −80 °C, the cell lysate was centrifuged at 4 °C for 10 min at 13,000× *g*. The supernatant was transferred to a new tube, and the protein concentration was determined using the Qubit Fluorometer and Quibit™ Protein Assay (Thermo Fischer Scientific, Waltham, WA, USA). Anti-HA antibody (GenScript, Piscataway, NJ, USA) was added to a ratio of 1 µg of antibody to 500 µg of total protein. After 1 h of incubation, the antibody–protein mix was transferred to a tube containing 100 µL of Dynabeads (Thermo Fisher Scientific, Waltham, WA, USA) and incubated for 1 h under rotation at 37 °C. The beads were then removed using a magnetic rack, and the supernatant was saved as the unbound fraction. The ASFV proteins were then eluted by adding 25 µg of HA peptide (GenScript, Piscataway, NJ, USA) in 100 µL 1X PBS to the beads. After 1 h of incubation, the beads were removed and discarded. All incubations were performed at RT in a rotator (Roto-Therm Plus, Ward’s Science, West Henrietta, NY, USA). The unbound and elution fractions were run in SDS-PAGE gels and evaluated by Western blot, as described above. 

### 2.10. Peptide Design and Synthesis

The complete amino acid sequence of the ASFV p30 was analyzed using the SnapGene Software (www.snapgene.com) and divided into 10 or 24 overlapping peptides of 24 or 12 amino acids in length, respectively, with four amino acids overlapping the next peptide up- and downstream. The peptide libraries were synthesized by GenScript ((≥85% purity) Piscataway, NJ, USA). Each peptide was diluted according to the manufacture’s recommendations and then aliquoted and stored at −20°C until use.

### 2.11. Enzyme-Linked Immunosorbent Assay (ELISA)

Purified recombinant ASFV pB602L, -p30, -pE184L, and -pI73R proteins were obtained by immunoprecipitation, as described above. Each protein was coated into Immunolon 1B polystyrene microtiter plates (Thermo Fisher Scientific, Waltham, WA, USA) at a concentration of 25 µg/mL in carbonate–bicarbonate solution (100 mM, pH 9.6) and incubated for 1 h at 37 °C. Following coating, the plates were washed three times with 300 µL of PBS and 0.05% Tween 20 per well. Blocking solution (1X PBS, 0.05% Tween 20, and 5% non-fat dry milk) was added to all wells, and the plates were placed at 4 °C overnight. The next day, the plates were washed three times, as above. Serial 2- or 3-fold dilutions of polled serum collected on days 0 and 35 p.im. (*Study 1*) or 0 and 42 p.im (*Study 2*) were diluted in blocking solution and added to coated and uncoated control wells (100 µL/well). Plates were incubated at RT for 1 h and washed three times, as described above. Goat Anti-Pig IgG-Fc fragment biotinylated antibody (Bethyl—Fortis Life Sciences, Waltham, WA, USA) diluted 1:4000 in blocking buffer was added to all wells (100 µL/well). Plates were incubated at 37 °C for 1 h and washed three times, as above. Pierce™ High Sensitivity Streptavidin-HRP (Thermo Fisher Scientific, Waltham, WA, USA) was diluted 1:4000 in blocking buffer and added to all wells (100 µL/well). Plates were incubated at 37 °C for 1 h and washed three times. Reactions were developed using TMB peroxidase substrate (SeraCare, Milford, MA, USA), and OD values were read at 450 nm, using the Synergy LX microplate reader (BioTek Instruments, Charlotte, VT, USA).

For the Pepscan ELISAs, each ASFV p30 peptide (24 or 12 aa) was diluted in carbonate–bicarbonate solution (100 mM, pH 9.6) at 25 µg/mL and coated onto Nunc^®^ Immobilizer™ Amino plates (Thermo Fisher Scientific, Waltham, WA, USA) overnight at 4 °C. After coating, the plates were washed three times, as described above. Blocking solution (1X PBS 0.05% Tween 20.5% non-fat dry milk) was then added to all wells, and the plates were placed at 37 °C for 1 h. After three washes, serial 2- or 3-fold dilution of polled serum from days 0 and 35 p.im. (Study 1) or 0 and 42 p.im (Study 2) were diluted in blocking solution and added to coated and uncoated control wells (100 µL/well). Plates were incubated at RT for one hour and then washed three times, as above. Anti-swine Fc biotinylated antibody (Bethyl—Fortis Life Sciences, Waltham, WA, USA) diluted 1:2500 in blocking buffer was added to all wells (100 µL/well). Plates were incubated at 37 °C for 1 h and washed three times. Pierce™ High Sensitivity Streptavidin-HRP (Thermo Fisher Scientific, Waltham, WA, USA) was diluted 1:4000 in blocking buffer and added to all wells (100 µL/well). Plates were incubated at 37 °C for 1 h and washed three times. Reactions were developed and read as described above.

### 2.12. Comparative Sequence Analysis of ASFV CP204L (p30) across ASFV Genotypes

Comparative sequence analysis of ASFV p30 was performed to assess sequence variability and identify areas of conservation between ASFV genotypes. Given that ASFV genotype classification is based on the ASFV *B646L* gene (encoding the major capsid protein p72), we first created a reference genotype dataset comprising 25 individual ASFV *B646L* sequences [38], which was used to classify full-length ASFV genome sequences available in GenBank. A total of 324 ASFV complete genome sequences (available in GenBank as of 12 January 2024) were retrieved and curated. Following removal of unverified, recombinant, and identical sequences (n = 32), the whole-genome dataset comprised 292 complete ASFV genomes. To classify these genomes into known ASFV genotypes, the *B646L* gene sequences were extracted from the whole-genome sequences and aligned with the reference *B646L* gene sequence dataset, using MAFFT [39]. Next, a maximum-likelihood phylogenetic tree was constructed, and sequences were classified within the known *B646L*-based genotypes. Subsequently, the *CP204L* gene sequences were extracted from the classified ASFV genomes and used to create a reference annotated *CP204L* dataset. All available *CP204L* sequences (n = 310, filtered with 50–100% similarity, 50–100 query coverage) were retrieved from GenBank (12 January 2024) and combined with the reference *CP204L* dataset to create a sequence alignment. Finally, a maximum likelihood phylogenetic tree was constructed to classify these *CP204L* gene sequences into different genotypes. Once all the *CP204L* sequences (n = 310, length = 585–606 bp) were classified phylogenetically into their respective genotypes, the nucleotide sequences were then translated into amino acid sequences and sequence diversity of p30 protein (length = 195–202 amino acid), and the immunogenic peptide 15 (Pep15, position = 120–131 aa) was analyzed. All the sequences were processed with Geneious Prime software, version 2024.0.

### 2.13. ASFV p30 3D Homology and Molecular Dynamics Simulation

The AFSV p30 protein structure model was generated using different structure prediction server tools, including Alphafold2 [40], I-Tasser [41], SWISS-model [42], and Phyre [43]. Given its limited sequence identity or similarity with existing structures, the initially generated p30 homology model was suboptimal. Therefore, the Alphafold2 model was selected and further refined via a molecular dynamics (MD) simulation. MD simulation was employed to explore the conformational dynamics of the p30 protein atoms within a well-defined hydration environment, providing an understanding of any deviations in the predicted protein structure over time. The interaction of the modelled p30 protein was analyzed using GROMACS (GROningen MAchine for Chemical Simulations) version 2023 and carried out for 1000-nanosecond (ns) simulations [44]. The modelled p30.pdb file generates protein topology using the 439a1 Amber force field. Afterwards, the p30 protein was solvated in a water box, followed by the addition of sodium (Na^+^) ions via the Genion tool to neutralize the system charge. Subsequently, the steepest descent with a conjugate gradient algorithm was used in the energy minimization process for 50,000 iteration steps, with a cutoff value of 1000 kJmol^−1^ that led to the stabilization of the p30 protein structure. Thereafter, the system was equilibrated in two phases, using the constant temperature (300 k), constant volume (NVT), constant temperature, and constant pressure (1 bar) (NPT) ensembles, with trajectories generated after every 2 femtoseconds (fs). The NVT phase was equilibrated with a constant number of particles (N), volume (V), and temperature (T) at 300 K, using the Parrinello–Rahman barostat pressure coupling method for 1 ns [45]; and the NPT phase was equilibrated with a constant number of particles (N), pressure (P), and temperature (T) at a pressure of 1.0 bar for 1 ns, using a Berendsen thermostat [46]. Lastly, a simulation was run to analyze the trajectories using GROMACS inbuilt tools. The analyses of simulations were performed and analyzed for the stability of the simulated protein by calculating the Root Mean Square Deviation (RMSD), Root Mean Square Fluctuation (RMSF), Radius of Gyration (Rg), and Solvent-Accessible Surface Area (SASA), using GROMACS inbuilt tools. For the protein structure representation, the MD simulated .pdb file format was examined using the PyMOL analysis tool [47].

### 2.14. Generation of Cell Line Stably Expressing ASFV p30 

To generate a cell line stably expressing the ASFV p30, the sequence of the *CP204L* gene was PCR amplified from the pUC57-ASFV-*CP204L* plasmid and subcloned into the lentiviral vector pSCALPS_Puro, a gift from Silvia Monticelli (Addgene plasmid #99636) [48]. To rescue lentiviral particles encoding ASFV p30, HEK 293T cells were co-transfected with pSCALPS_Puro-ASFV-*CP204L* and the packaging plasmids, psPAX2 and pMD2.G, gifts from Didier Trono (Addgene plasmid # 12260 and Addgene plasmid # 12259, respectively), using Lipofectamine 3000 reagent (Thermo Fisher Scientific, Waltham, MA, USA). At 48 h post-transfection, the supernatant containing the lentivirus was collected and used to transduce BHK21 cells. Cells were cultured in 6-well plates (5 × 10^5^ cells per well) and transduced with 500 µL of the lentivirus suspension collected from the packaging HEK 293T cells and incubated at 37 °C for 2 h, with gentle rocking, each 15 min. After adsorption, 1.5 mL of complete MEM was added to the cells. At 48 h post-transduction, the cell culture medium was replaced by complete MEM containing 4 µg/mL of puromycin dihydrochloride (Thermo Fisher Scientific). The BHK21-ASFVp30 stable cells were kept in puromycin selection for six passages, and the expression of ASFV p30 was assessed by Western blot and IFA. 

### 2.15. ADCC Assay

To determine if the antibodies generated against ASFV p30 were capable of inducing antibody-dependent cell cytotoxicity (ADCC), the BHK21-ASFVp30-stable cells (target cells) were seeded in 96-well plates (2.5 × 10^4^ cells per well), and 24 h after plating, the cell culture medium was removed, and the cells were incubated with different concentrations of pooled serum from days 0 and 35 p.im. (Study 1) diluted in serum-free RPMI. After 3 h of incubation, the cells and bound antibodies were overlayed with naïve swine PBMC (effector cells; 1 × 10^5^ cells per well) diluted in complete RPMI (Corning Inc., Corning, NY, USA) 20% FBS, at a 1:4 target/effector cell ratio. The supernatant of the cells and appropriated controls (low (complete RPMI 10% FBS media) and high controls (0.1% triton X-RPMI media)) were collected at 72 h, and the release of lactate dehydrogenase was measured using the Cytotoxicity Detection Kit (LDH) (Roche, Basel, Switzerland), following the manufacturer’s instructions. The percentage of cytotoxicity was determined by subtracting the OD values of the samples minus the assay’s low control, divided by the OD value of the high control minus low control, and this number was multiplied by 100 (($\frac{Sample\;OD-Low\;control\;OD}{High\;control\;OD-Low\;control\;OD})\times 100$) (490 nm, Synergy LX microplate reader (BioTek Instruments, Charlotte, VT, USA)). Percent cytotoxicity between days 0 and 35 p.im. were compared. 

### 2.16. Statistical Analysis

All in vitro testing was performed in triplicates. Statistical analysis was performed using GraphPad Prism software version 10.2.3 (Boston, MA, USA). All assays were performed in triplicate. The data are shown as mean ± SEM, and *p*-values of <0.05 were considered statistically significant.

## 3. Results

### 3.1. Generation of ORFV-ASFV Expression Vectors 

**Figure 1:**
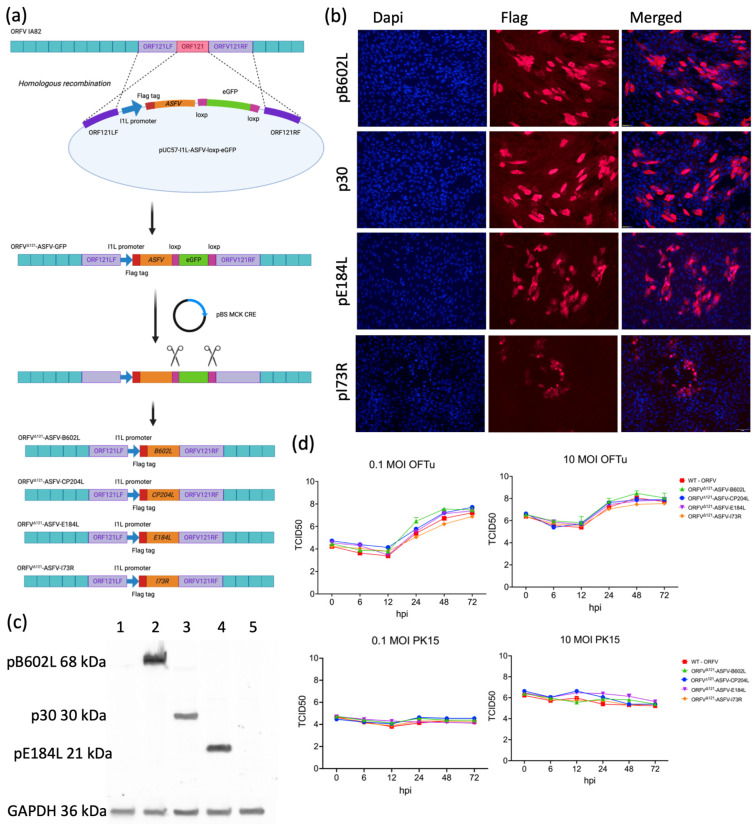
Construction and characterization of ORFV recombinants. (**a**) Schematic representation of homologous recombination between plasmid pUC57-I1L-ASFV-loxp-eGFP and ORFV-IA82 genome. The recombinant viruses were passed in Cre recombinase-expressing cells for GFP removal. (**b**) Immunofluorescence assay performed in OFTu cells infected with different ORFV recombinants. Cells were fixed and incubated with monoclonal mouse Anti-FLAG^®^ M2 antibody, followed by incubation with goat anti-mouse IgG secondary antibody (Alexa Fluor^®^ 594 conjugate). Nuclear staining was performed with the DNA-specific stain DAPI. (**c**) Western blot assay performed in OFTu cells infected with ORFV-IA82 (1), ORFV-^∆121^-ASFV-B602L (2), ORFV-^∆121^-ASFV-CP204L (3), ORFV-^∆121^-ASFV-E184L (4), and ORFV-^∆121^-ASFV-I73R (5); GAPDH was used as the loading control. One hundred micrograms of whole-cell lysate was resolved by SDS-PAGE in 10% acrylamide gel and transferred to a nitrocellulose membrane, probed with monoclonal mouse Anti-FLAG^®^ M2 antibody and Proteintech Rabbit GAPDH antibody, followed by IRDye^®^ 800CW Mouse IgG Secondary antibody and IRDye^®^ 680RD Rabbit IgG Secondary antibody. (**d**) Single-step (0.1 MOI) and multi-step (10 MOI) growth curves of ORFV recombinants. OFTu and PK15 cells were infected and virus titers were collected at 0, 6, 12, 24, 48, and 72 h post-infection. The virus titers were determined by Spearman and Karber’s method and expressed as tissue culture infection dose 50 per mL (TCID_50_/mL).

The ORFV^∆121^ASFV-*B602L*, -*CP204L*, -*E184L*, and -*I73R* recombinant viruses were successfully generated by homologous recombination, and purified recombinant viruses were selected based on the expression of the GFP reporter (Figure 1a). Following infection/transfection, a minimum of ten independent GFP-positive clones of each virus were collected on plaque assay one. At each subsequent plaque assay, three independent clones were selected, one of which was used for the following round of plaque assay. After five or seven rounds of selection via plaque assay, wild-type plaques (no GFP expression) were no longer observed. Then, OFTu cells were infected with each GFP-expressing clone, and DNA was extracted from cells and supernatant 72 h post-infection. The purification of each clone was verified by PCR, confirming the insertion of the ASFV gene and complete deletion of ORFV *ORF121*.

The GFP reporter was removed from recombinant viruses using Cre-lox recombination. Purified ORFV^Δ121^-ASFV clones were inoculated and passaged in Cre recombinase expressing OFTu cells (three times) and purified by five rounds of plaque assay, with GFP-negative plaques being selected and used for the next round of plaque purification. The successful removal of the GFP reporter gene from the recombinant virus genomes was confirmed by PCR. The expression of pB602L, p30, pE184L, and pI73L by the recombinant viruses was confirmed by IFA and Western blot assays (Figure 1b and Figure 1c, respectively). After purification and confirmation of recombinant ASFV protein expression, the ORFV^∆121^ASFV-*B602L*, -*CP204L*, -*E184L*, and -*I73R* recombinant viruses were subjected to complete genome sequencing. The results confirmed the correct insertion of the ASFV genes into the *ORFV121* locus and complete removal of the GFP reporter gene (BioProject ID PRJNA1083465). 

### 3.2. In Vitro Characterization of ORFV^Δ121^-ASFV Recombinants

The replication kinetics of ORFV^∆121^ASFV-*B602L*, -*CP204L*, -*E184L*, and -*I73R* recombinant viruses were compared to the wild-type ORFV strain IA82 in OFTu and PK15 cells using one-step and multi-step growth curves. Cells infected with 0.1 and 10 MOI of each virus were harvested at 0-, 6-, 12-, 24-, 48-, and 72-hours p.i., and viral titers were determined at each time point. As shown in Figure 1d, all recombinant viruses replicated to similar titers as the WT strain in the natural host OFTu cells. However, the replication of all recombinants and WT was impaired in PK15 cells (Figure 1d).

**Figure 2:**
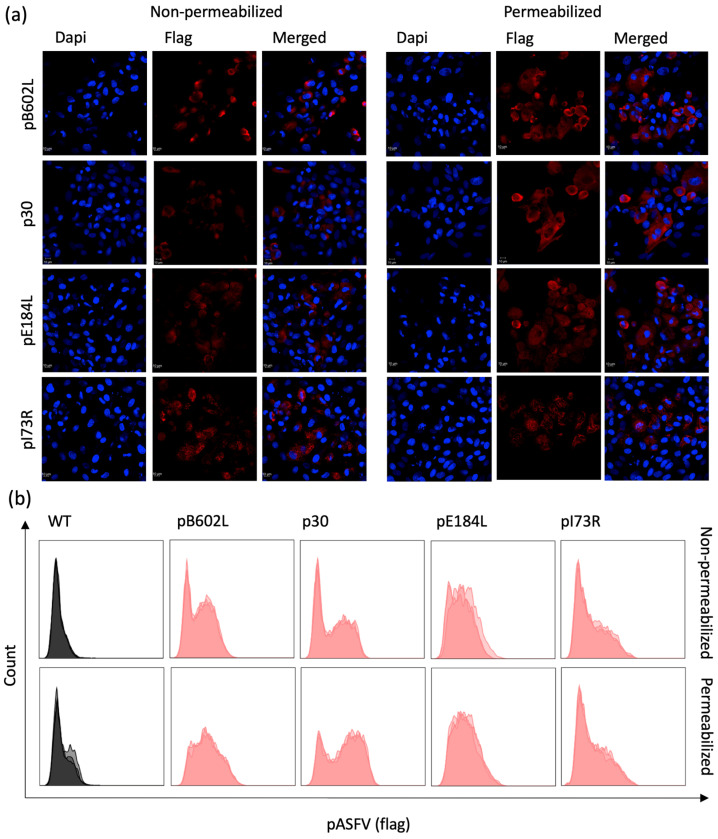
Localization of ASFV proteins in cells infected with ORFV recombinants. (**a**) Immunofluorescence assay was performed in permeabilized and non-permeabilized OFTu cells infected with 1 MOI of the different ORFV recombinants. Cells were fixed and incubated with monoclonal mouse anti-FLAG^®^ M2 antibody, followed by goat anti-mouse IgG secondary antibody (Alexa Fluor^®^ 594 conjugate). Nuclear staining was performed with the DNA-specific stain DAPI. Cells were analyzed with a Zeiss 710 Confocal microscope. (**b**) Flow cytometry of permeabilized and non-permeabilized OFTu cells infected with 5 MOI of the different ORFV recombinants. Cells were stained with monoclonal mouse Anti-FLAG^®^ M2 antibody, followed by goat anti-mouse IgG secondary antibody (Alexa Fluor^®^ 488 conjugate), and analyzed with the Attune NxT cytometer. Panel was made using the FlowJo software v10.10 layout tool.

To characterize the expression profile of the ASFV pB602L, p30, pE184L, and pI73R proteins by the recombinant ORFV^∆121^ASFV-*B602L*, -*CP204L*, -*E184L*, and -*I73R* viruses, we used IFA and flow cytometry assays. OFTu cells were infected with each ORFV^∆121^-ASFV recombinant viruses (MOI=5) and harvested at 48 h p.i. ASFV pB602L, p30, and pE184L were detected by IFA and flow cytometry in permeabilized and non-permeabilized cells (Figure 2a and Figure 2b, respectively). The fact that all proteins were detected in non-permeabilized cells in both IFA and flow cytometry assays suggest that the ASFV pB602L, p30, pE184L, and pI73R proteins are expressed at the cell membrane of OFTu cells infected with the recombinant ORFV^∆121^-ASFV viruses. 

### 3.3. ASFV p30 Elicits Robust Antibody Responses following ORFV-ASFV Immunization 

**Figure 3:**
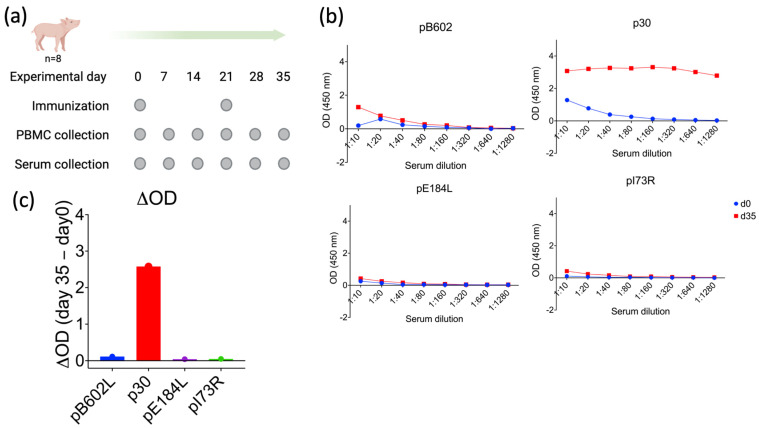
Evaluation of the immunogenicity of ASFV proteins delivered by a pool of ORFV recombinants. (**a**) Eight four-week-old piglets were immunized with a cocktail of ORFV recombinants on days 0 and 21. Serum samples were collected weekly until day 35 (Study 1). (**b**) ASFV-specific antibodies levels were evaluated by ELISA on pooled samples from 0 dpi and 35 dpi. (**c**) The difference in OD values obtained by ELISA in serum samples from 0 dpi and 35 dpi.

**Figure 4:**
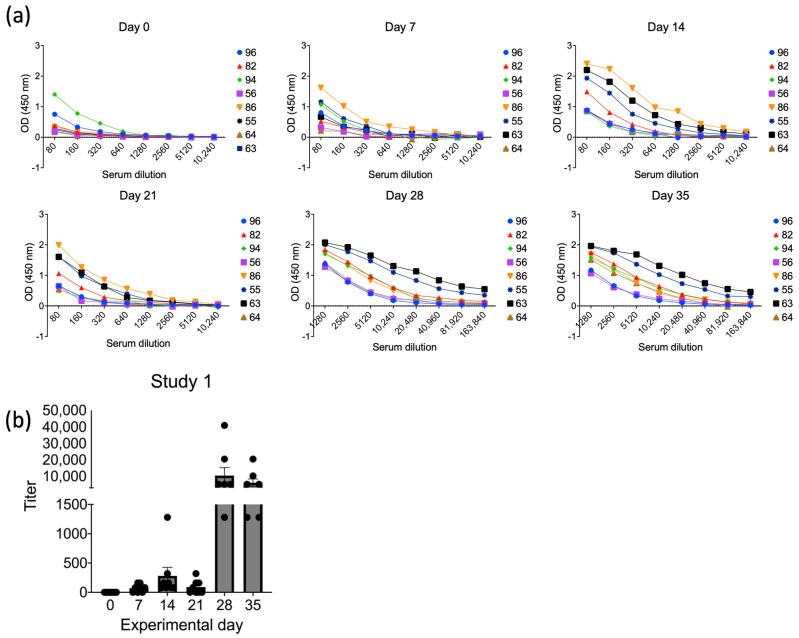
Antibody responses elicited by ASFV p30. (**a**) ELISA results for individual animals at all experimental timepoints; numbers represent the identification of each pig. (**b**) Endpoint titers elicited by ASFV p30 in piglets immunized with ORFV-^∆121^-ASFV-CP204L in all experimental timepoints. The endpoint is defined as the reciprocal of the highest serum dilution that gives a reading above the cutoff, calculated as mean OD of negative samples plus 3 times the standard deviation.

The antibody responses elicited against ASFV pB602L, p30, pE184L, and pI73R were evaluated using ELISA assays following the immunization of pigs with the ORFV^∆121^ASFV-*B602L*, -*CP204L*, -*E184L*, and -*I73R* recombinant viruses. Pooled serum samples collected on day 0 and 35 p.im. (Study 1, Figure 3a) were tested against recombinant purified ASFV pB602L, p30, pE184L, and pI73R proteins using ELISAs. As shown in Figure 3b, while only low antibody binding was detected against ASFV-pB602L, pE184L, and pI73R, robust antibody binding was detected against ASFV p30, as evidenced by a high ΔOD between day 35 and day 0 p.im. (OD day 35–OD day 0) (Figure 3c). The dynamics of antibody responses against p30 in ORFV^∆121^ASFV-immunized pigs were also evaluated by ELISA, using serum samples from individual animals in Study 1. As shown in Figure 4a,b, increasing p30 antibody titers were detected starting on day 14 p.im., with a robust and anamnestic antibody response being detected on day 28 p.im. (a week after the booster immunization on day 21, Study 1) (Figure 4b). 

**Figure 5:**
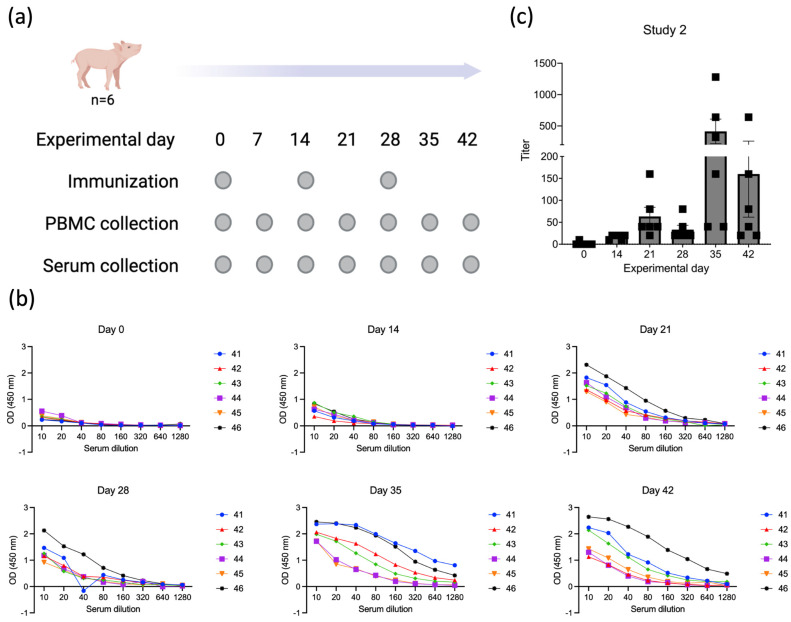
Evaluation of the immunogenicity of ASFV p30 delivered by ORFV-^∆121^-ASFV-CP204L. (**a**) Six four-week-old piglets were immunized with ORFV-^∆121^-ASFV-CP204L at days 0, 14, and 28 p.im. Serum and PBMC samples were collected on days 0, 14, 21, 28, 35, and 42 p.im. (Study 2). (**b**) ELISA results for individual animals at all experimental timepoints; numbers represent the identification of each pig. (**c**) Endpoint titers elicited by ASFV p30 in piglets immunized with ORFV-^∆121^-ASFV-CP204L in all experimental timepoints. The endpoint is defined as the reciprocal of the highest serum dilution that gives a reading above the cutoff, calculated as mean OD of negative samples plus 3 times the standard deviation.

To confirm the immunogenicity of p30, we performed a second immunization study (Study 2, Figure 5a) in which six four-week-old piglets were immunized only with the recombinant ORFV^∆121^ASFV-*CP204L*. Prime immunization was performed on day 0, and boosters were given on days 14 and 28 p.im. Serum samples were collected on days 0, 14, 21, 28, 35, and 42 p.im. Similar to what we observed in Study 1, all animals in Study 2 started showing detectable antibody titers against p30 on day 14 p.im. (Figure 5b,c). As expected, an increase in antibody titers was observed on day 21 p.im. following the booster on day 14. An anamnestic antibody titer increase was observed on day 35 p.im., following the second booster immunization on day 28 (Figure 5b,c). 

### 3.4. Identification of Immunodominant Linear B-Cell Epitope in ASFV p30

**Figure 6:**
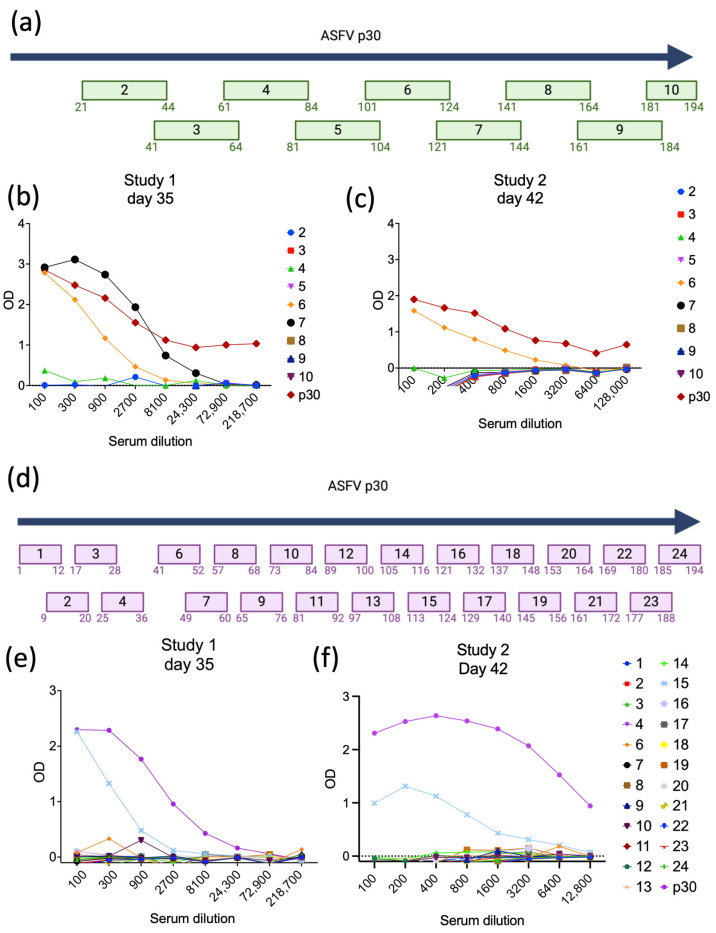
Identification of B-cell epitopes of ASFV p30. (**a**) The full-length ASFV p30 amino acid sequence was divided into peptides of 24 amino acids of length, with the last four amino acids overlapping the next peptide. (**b**) Antibodies against each peptide in serum from piglets collected at 35 dpi (Study 1) and (**c**) 42 dpi (Study 2). (**d**) The full-length ASFV p30 was further divided into peptides of 12 amino acids of length, with the last four amino acids overlapping the next peptide. (**e**) Pepscan ELISA detecting antibodies against each peptide in serum from piglets collected at day 35 p.im. (Study 1) and (**f**) day 42 p.im. (Study 2).

To identify potential linear B-cell epitopes within the ASFV p30 coding sequence, initially we analyzed and divided the sequence of p30 into 10 overlapping peptides comprising 24 amino acids in length (four amino acids overlapping with the next peptide) (Figure 6a). We then performed Pepscan ELISA assays with the individual peptides, using pooled serum samples collected from immunized pigs in Studies 1 and 2. As shown in Figure 6b,c, strong antibody binding was detected with peptides 6 and 7 (Pep6, 101-SENIHEKNDNETNECTSSFETLFE-124; and Pep7 121-TLFEQEPSSEVPKDSKLYMLAQKT-144) when pooled serum samples from day 35 p.im. from Study 1 were tested against the peptide library in the ELISAs (Figure 6b). Notably, testing of pooled serum samples from day 42 p.im. from Study 2 confirmed the high immunogenicity of Pep6, as evidenced by strong antibody binding against Pep6 in the Pepscan ELISA (Figure 6c), whereas no antibody binding was detected for Pep7.

To refine the epitope mapping of ASFV p30, we designed a peptide library in which the full-length coding sequence of ASFV p30 was divided into 24 smaller peptides of 12 amino acids in length, with each peptide overlapping the next peptide by 4 amino acids on each side (Figure 6d). This peptide library was then tested using Pepscan ELISAs and pooled serum samples from immunized pigs in Study 1 (day 35 p.im.) and Study 2 (day 42 p.im.) Notably, the results of the Pepscan ELISAs demonstrated strong antibody binding to peptide 15 (Pep15, 113-NECTSSFETLFE-124), which overlaps entirely with the immunogenic Pep6 described above (Figure 6b,c). These results demonstrate that Pep15 constitutes a highly immunogenic linear B-cell epitope (from hereafter called *Ep15*) within the ASFV p30 protein. 

### 3.5. The Immunodominant p30 Epitope 15 Is Conserved among ASFV Genotypes

**Figure 7:**
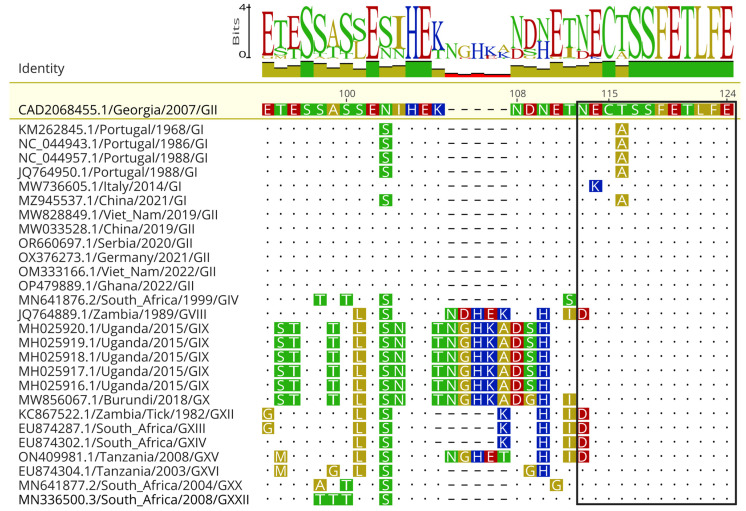
Sequence alignment of 28 ASFV p30 sequences from genotypes I, II, IV, VIII, IX, X, XII, XIII, XIV, XV, XVI, XX, and XXII available on GenBank shows that epitope 15 is highly conserved among ASFV genotypes. The region immediately upstream of epitope 15 presents with several mutations and deletions. GI, genotype I; GII, genotype II; GIII, genotype III; GIV, genotype IV; GV, genotype V; GVI, genotype VI; GVII, genotype VII; GVIII, genotype VIII; GIX, genotype IX.

A comparative sequence analysis was performed with ASFV p30 sequences available on GenBank (n = 526, as of 12 January 2024) to determine the overall conservation of the full-length protein and of the immunodominant *Ep15* between distinct ASFV genotypes. Overall, the amino acid sequence identity among complete p30 sequences ranged from 86 to 100%. There is a five amino acid HEKND insertion at position 104 in GVIII and GXV, and a HETNG insertion in the same position in GIX and GX; however, this insertion is not present in GI, GII, GIV, GXII, GXIII, and GXIV (Figure 7). The intragenotypic sequence identity of complete p30 sequences was 99–100%. Notably, the region comprising *Ep15* (113-NECTSSFETLFE-124) was highly conserved (100%) among GII, GIX, and GX isolates (Figure 7), while a N113D substitution was observed in GVIII, GXIII, GXIV, and GXV ASFV isolates; and a T116A substitution was observed in contemporary and historical GI viruses (Figure 7). There are five additional sequences of “low-virulence ASFV” in the GenBank (GenBank accession KM262845.1, NC_044943.1, NC_044957.1, JQ764950.1, and MZ945537.1) with T116A substitution in *Ep15* [49]. Another GI isolate (GenBank accession MW736605.1) has a E114K substitution (Figure 7).

### 3.6. Structural Analysis of ASFV p30 Protein Predicts That Ep15 Is Located within an Exposed Loop

**Figure 8:**
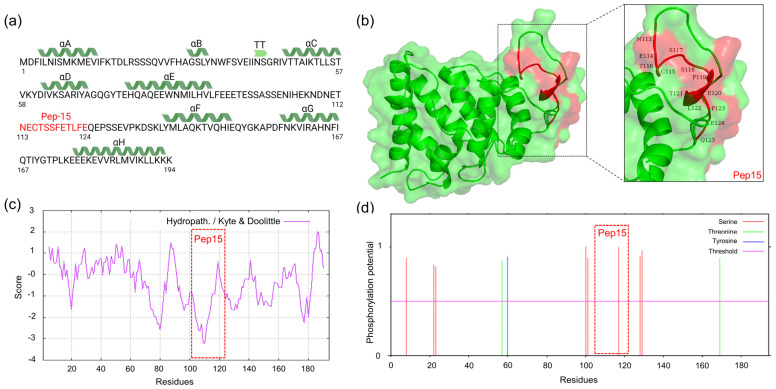
Modeling and structural analysis of ASFV p30. (**a**) The 2D structure of the p30 protein, with Pep15 amino acid residues highlighted in red, showing the alpha helices labeled from A to H in green, while turns are depicted as TT in light green arrow shape. (**b**) Surface and cartoon presentation of the p30 proteins (green), with Pep15 amino acid residues, are highlighted in the zoom-in box. The amino acid residues containing Pep15 are colored red, with proper one-letter residue labelling to indicate the positions within the sequence. (**c**) Hydrophobicity prediction for p30 protein using Expasy ProtScale displayed via the Hydropathy/Kyte and Doolittle plot. (**d**) Predicted phosphorylation sites on the p30 protein, with the highest score observed at Ser117, identified using the NetPhos-3.1 online server tool.

The p30 protein comprises eight α-helices designated αA-αH (Figure 8a). Notably, it harbors a highly flexible loop from amino acid residues Phe91 to Ser135, between the αE and αF secondary structures (Figure 8b). Our in silico analysis revealed that the immunogenic Ep15 identified as an immunodominant B-cell epitope within p30 is located in the flexible loop region (Figure 8b). The loop region of p30, especially the Ep15 amino acid residues, is predicted to exhibit a highly hydrophilic nature, enriched with charged aminos that are likely to be exposed on the surface of the protein (Figure 8c). The Ep15 contains putative phosphorylation sites, with Ser117 residues having the highest predicted score (0.990) (Figure 8d). 

To refine the structural prediction of ASFV p30, we used molecular dynamics (MD) simulations. Molecular dynamics simulations were analyzed based on the Root Mean Square Deviation (RMSD), Radius of Gyration (Rg), Root Mean Square Fluctuation (RMSF), and Solvent-Accessible Surface Area (SASA) values throughout the 1000 ns simulation (Supplementary Figure S2). The RMSF analysis revealed atom fluctuation starting at amino acid residues L107-C117 during the simulation, with an average RMSF value of 0.9 nm, highlighting the structural flexibility of the protein loop region in comparison to the overall structure of p30 protein containing the stable secondary structure of an α-helix region (Supplementary Figure S2D). Within this loop region, between two α-helixes, Thr112 and Asn113 achieve the maximal RMSF value equal to 1.1 nm. As shown in the predicted ASFV p30 structure (Figure 8b), the loop region seems to be exposed on the surface of the protein, being accessible to solvent molecules. Notably, an overlay of the immunogenic Ep15 sequence with the predicted structure of ASFV p30 revealed that Ep15 is in the exposed loop region of the protein (Figure 8b). 

### 3.7. Antibodies against ASFV p30 Elicit Antibody-Dependent Cellular Cytotoxicity (ADCC)

**Figure 9:**
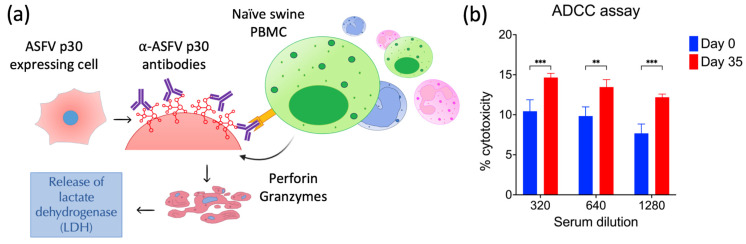
Antibodies against ASFV p30 elicit antibody-dependent cell cytotoxicity. (**a**) ADCC representation: Antibodies bind to p30 expressed on the cell surface. The Fc portion of the antibodies is recognized by the Fc receptor of cytotoxic lymphocytes (e.g., natural killer cells), which trigger the release of perforins and other enzymes that lead to the death of the target cell. (**b**) Analysis of ADCC through measure of lactate dehydrogenase in the supernatant of cells overexpressing ASFV p30 incubated with serum from 0 dpi and 35 dpi and overlaid with naïve swine PBMC. ** *p* < 0.001; *** *p* < 0.0001.

The function of ASFV p30-specific antibodies was assessed by ADCC assay. For this, BHK21 cells stably expressing ASFV p30 (target cells) were incubated with dilutions of pooled serum from days 0 and 35 post-immunization (Study 1). Subsequently naïve swine PBMCs (effector cells) were added to the antibody-cell complexes. Lactase dehydrogenase release was determined in the supernatant of each well as a measure of cellular cytotoxicity. As shown in Figure 9b, significantly higher cellular cytotoxicity was observed in the supernatant of cells exposed to ASFV p30 positive serum (day 35 p.im.) when compared to cells exposed to serum from day 0. These results suggest that antibodies against ASFV p30 mediate ADCC.

## 4. Discussion

Here, we assessed the immunogenicity of ASFV proteins expressed by an ORFV vector. We generated four ORFV recombinants by homologous recombination, replacing the *ORFV121* with ASFV genes *B602L*, *CP204L*, *E184L*, and *I73R*. All recombinant viruses successfully expressed the pB602L, p30, pE184L, and pI73R proteins under the early poxvirus promoter I1L [34]. Deletion of *ORFV121* and its replacement with ASFV proteins did not affect the ability of the recombinant viruses to infect and replicate in vitro, as their replication kinetics were similar to the wild-type ORFV strain in primary OFTu cells. As expected, virus replication was markedly impaired in swine cells (PK15). This was previously described in primary swine turbinate cells [50,51,52], indicating that ORFV viruses do not, or only minimally, replicate in cells of swine origin, which constitutes a safety feature for the use of ORFV-based vectors in swine. 

Next, we analyzed whether the ASFV proteins were expressed in the surface of ORFV-ASFV-infected cells. All four ASFV proteins—pB602L, p30, pE184L, and pI73R—were detected in both permeabilized and non-permeabilized cells by IFA and flow cytometry, indicating that these proteins are being trafficked and expressed at the cell membrane of ORFV^∆121^ASFV-infected cells. 

After the immunization of piglets with a cocktail of ORFV-ASFV recombinants (ORF-^∆121^-ASFV-*B602L*, -*CP204L*, -*E184L*, and -*I73R*), we detected a strong antibody response against ASFV p30 (gene *CP204L*) in immunized animals. The immunogenicity of ASFV p30 was confirmed in a second experiment, in which piglets were immunized only with ORF-^∆121^-ASFV-*CP204L* recombinant virus. Similar to what we observed in Study 1, animals in Study 2 also developed high antibody titers against ASFV p30, albeit overall titers were lower than those detected in animals in Study 1. As observed in other immunization studies with ORFV vectors in swine [50,51,52,53,54,55], an anamnestic antibody response to ASFV p30 was detected a week after the booster immunizations on day 21 (Study 1) or 28 (Study 2), respectively.

The role of antibodies in protection against ASFV is not fully understood; however, many studies have shown the association of neutralizing antibodies and protection against challenge. Pigs that received anti-ASFV immunoglobulin survived homologous challenge and presented with no clinical signs of the disease [56]. One piglet born from a convalescent sow survived the ASFV challenge, with no display of clinical signs or viremia, and this was attributed to passive antibodies acquired via colostrum [57]. More recently, a study in inbred pigs demonstrated that survival to ASFV challenge weakly correlates to antibody responses, and protection seems to have a stronger association with T-cell responses [58]; however, the presence of neutralizing antibodies was associated with protection in animals vaccinated with live attenuated vaccine candidates [59]. The results here demonstrate the immunogenicity of ASFV p30 and its ability to elicit robust antibody responses following immunization in swine. The exact function of p30-specific antibodies for ASFV protection, however, remains to be elucidated. 

Because of its high immunogenicity, ASFV p30 has been included in several challenge trials in pigs; however, the results of these trials are conflicting. Barderas and colleagues [60] found that the immunization of pigs with chimeric p54/p30 protein resulted in the survival of animals after challenge, and remarkably lower viremia compared to control animals. High titers of ASFV p30-specific antibodies were also detected in inbred and outbred pigs that recovered from a low-virulent challenge with strain OURT88/1, indicating that these antibodies may contribute to the reduction of clinical signs and viremia against this strain [58]. On the other hand, neutralizing antibodies elicited in animals immunized with a pool of proteins, including ASFV p30, did not result in protection, and specific neutralizing antibodies against p30 were also shown not to be sufficient to protect against virulent ASFV challenge [61,62].

Antibody-dependent cell cytotoxicity (ADCC) has been described in ASFV infection. The activity of neutrophils and porcine bone marrow cells as effector cells was confirmed utilizing anti-sera from different strains of ASFV, but the specificity of the antibodies that recruited effector cells to induce ADCC was not determined [63,64]. The first association between ADCC and reduced ASFV viremia emerged from a series of animal challenge experiments [65]. The results here show that ASFV p30-specific antibodies may contribute to the recruitment of cytotoxic cells and the elimination of p30-expressing cells. Since ASFV p30 is expressed in the surface of ASFV-infected cells [30], this effector function of p30 specific antibodies may contribute to the elimination and/or clearance of ASFV infected cells in vivo. In addition to the effector ADCC function of p30 antibodies described for the first time here, previous studies have shown that p30 antibodies also neutralize ASFV entry and internalization in porcine macrophages and Vero cells in vitro. However, they are not sufficient to provide complete immune protection against ASFV in vivo [62].

To characterize and identify immunogenic B-cell epitopes within ASFV p30, we designed libraries of overlapping peptides covering the full-length sequence of the protein and used them in Pepscan ELISAs. Initial screening using 24 aa long peptides identified Pep6, 101-SENIHEKNDNETNECTSSFETLFE-124, as immunogenic, as evidenced by strong antibody binding in the Pepscan ELISA. Subsequent screening of a library of smaller overlapping peptides of 12 aa in length further refined our epitope mapping, identifying Pep15 113-NECTSSFETLFE-124 (which is entirely embedded within Pep6) as an immunodominant linear B-cell epitope within p30. The immunogenicity of ASFV p30 oligopeptides (aa 91–130 and 111–160) containing Ep15’s sequence has been described previously in pigs immunized with a recombinant alphavirus carrying ASFV p30 and boosted with the low-virulence genotype I strain OURT88/3 or infected with ASFV OURT88/3 only [66]. The residues 121-TLFE-124 are also part of a larger oligopeptide identified as immunogenic through the screening of two monoclonal antibodies against ASFV p30. This study also identified a large immunogenic oligopeptide between amino acids 61 and 93 of ASFV p30 [67]. Recently, an immunogenic seven-residue peptide (HNFIQTI) localized downstream of Ep15 was also identified through screening of monoclonal antibodies [68].

A comparative sequence analysis of ASFV p30 revealed that the immunogenic Ep15 is highly conserved across different genotypes which have evolved over decades. For example, on ASFV genotype II viruses, which make nearly 24 phylogenetic clusters, Ep15 is 100% conserved among these isolates. Within ASFV genotype I viruses, which are also currently circulating in Asia, a few amino acid substitutions were observed. Sequences with T116A substitution are observed in genotype I ASFV viruses [49,69]. Additionally, E114K mutation was reported in ASFV genotype I isolates from Sardinia, Italy, in 2014 [70]. The fact that Ep15 is 100% conservation among numerous genotypes of ASFV may enable the development of improved detection and control tools for ASFV. The early antibody response against ASFV p30, along with our findings demonstrating the immunodominance of Ep15, can facilitate the development of differentiating infected from vaccinated animal (DIVA)-compatible vaccine candidates by utilizing Ep15 as a marker for future ASFV vaccine candidates.

Here, we demonstrated the immunogenicity of an ORFV-^∆121^-*CP204L* recombinant virus expressing the ASFV p30 protein. Upon immunization with ORFV-^∆121^-ASFV-*CP204L* virus, immunized animals developed high antibody titers against ASFV p30 (gene *CP204L*), which was consistent with the high titer of ASFV p30 antibody production seen in ASFV infections. We showed that antibodies against ASFV p30 are involved in the development of ADCC and, thus, may contribute to the clearance of virus infected cells. Furthermore, we identified one linear epitope of 12 aa in length (Ep15) within ASFV p30 that appears to be highly immunogenic. This epitope is conserved among various ASFV genotypes, and it appears to be a good candidate for a marker in the development of DIVA vaccines. The results presented in this study contribute to a better understanding of the role of ASFV p30 in viral immunity and provide insights for the generation of a safe and effective vaccine against ASF.

## Data Availability

The raw sequence files of Orf recombinants generated in this study are available at BioProject, under project ID PRJNA1083465.

## Supplementary Materials

The following supporting information can be downloaded at https://www.mdpi.com/article/10.3390/v16050758/s1, Figure S1: Characterization of BHK21 cells stably expressing ASFV p30, Figure S2: Molecular dynamics simulation of ASFV p30 structure.
