# Supplementary material for: Identification of an Immunodominant B-Cell Epitope in African Swine Fever Virus p30 Protein and Evidence of p30 Antibody-Mediated Antibody Dependent Cellular Cytotoxicity
Source: Viruses. 2024 May 10;16(5):758. doi: 10.3390/v16050758 (PMC11125664; doi:10.3390/v16050758)
Supplement: Supplementary file 1 [file viruses-16-00758-s001.zip › viruses-2931828-supplementary.pdf]

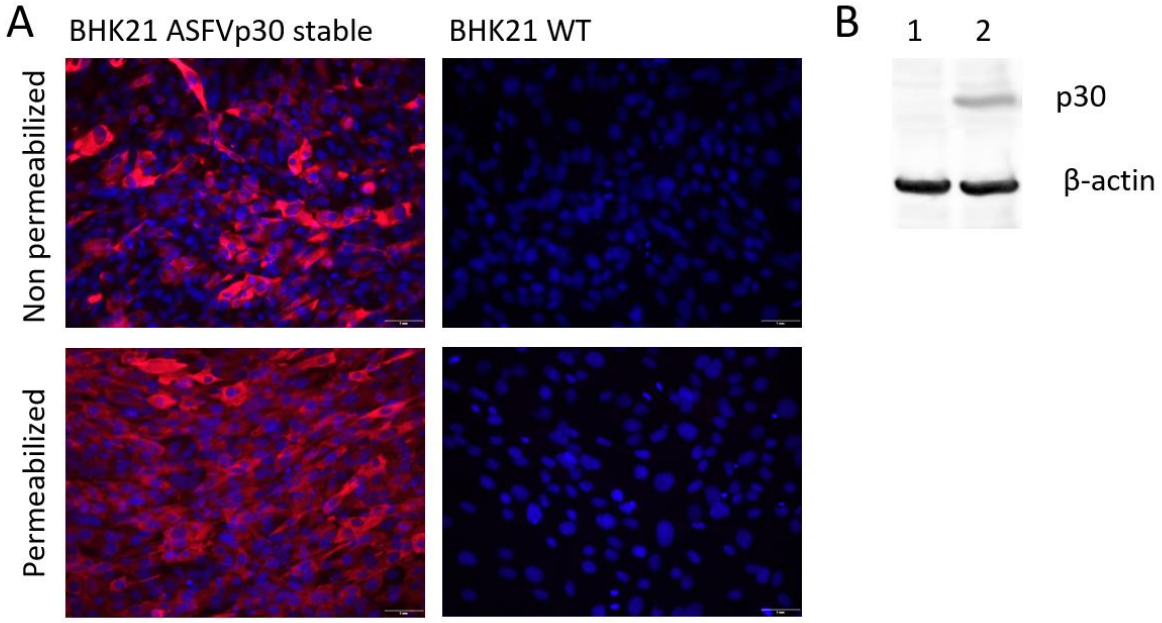

Supplemental Figure S1. Characterization of BHK21 cells stably expressing ASFV p30. (A) Immunofluorescence assay was performed in permeabilized and non-permeabilized BHK21 ASFVp30 stable cells. Cells were fixed and incubated with monoclonal mouse anti-ASFVp30 clone 60.11.6 antibody (generated in house) followed by goat anti-mouse IgG secondary antibody (Alexa Fluor® 594 conjugate). Nuclear staining was performed with the DNA specific stain DAPI. (B) Western blot assay was performed in BHK21 wild type (1) and BHK21 ASFVp30 (2) stable cells. One hundred micrograms of whole cell lysate were resolved by SDS-PAGE in 10% acrylamide gel and transferred to a nitrocellulose membrane, probed with monoclonal mouse anti-ASFVp30 clone 60.11.6 antibody (generated in house) with monoclonal mouse Anti-FLAG® M2 antibody and Santa Cruz Rabbit -actin antibody followed by IRDye® 800CW Mouse IgG Secondary antibody and IRDye® 680RD Rabbit IgG Secondary antibody.

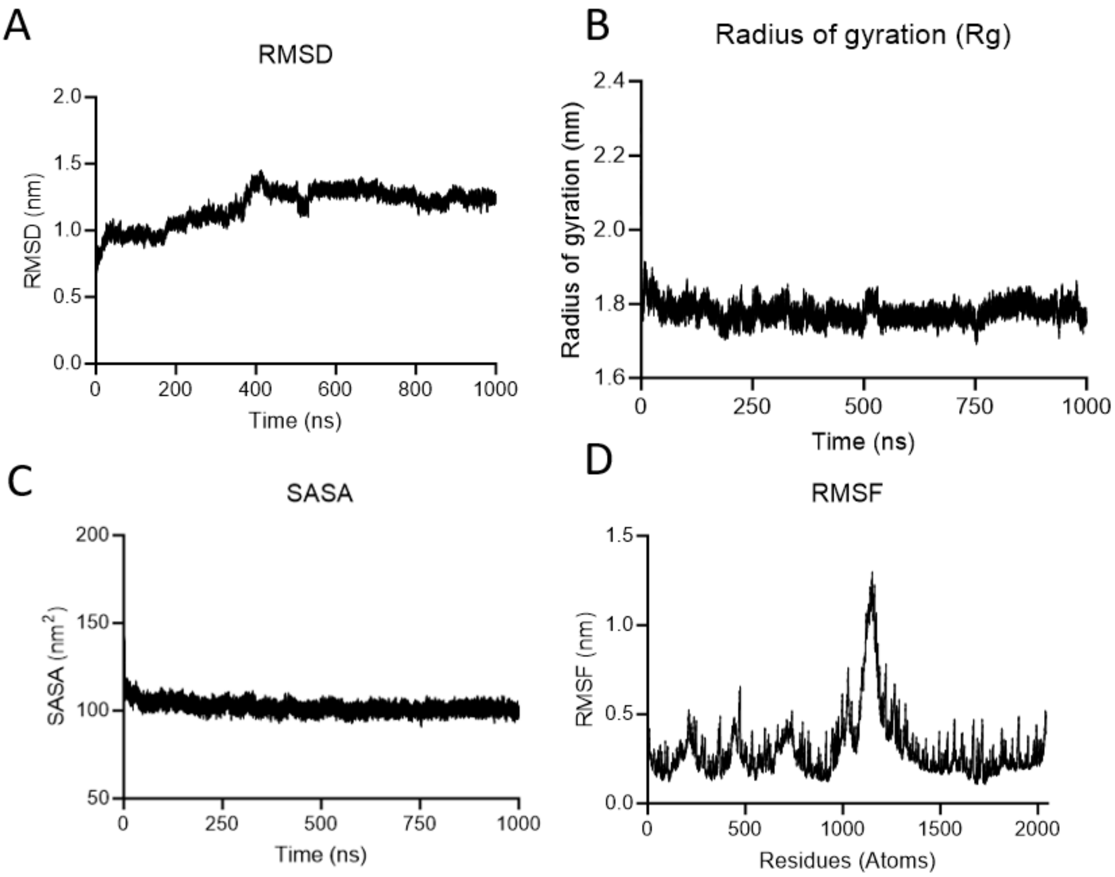

Supplemental Figure S2. Molecular dynamics simulation of ASFV p30 structure. (A) Root-mean-square deviation (RMSD). (B) Radius of gyration (Rg). (C) Root-mean-square fluctuation (RMSF). (D) solvent-accessible surface area (SASA).
